# Actinomycetes from the Red Sea Sponge *Coscinoderma mathewsi*: Isolation, Diversity, and Potential for Bioactive Compounds Discovery

**DOI:** 10.3390/microorganisms8050783

**Published:** 2020-05-23

**Authors:** Yara I. Shamikh, Aliaa A. El Shamy, Yasser Gaber, Usama Ramadan Abdelmohsen, Hashem A. Madkour, Hannes Horn, Hossam M. Hassan, Abeer H. Elmaidomy, Dalal Hussien M. Alkhalifah, Wael N. Hozzein

**Affiliations:** 1 Department of Microbiology and Immunology, Nahda University in Beni Suef, Beni-Suef 65211, Egypt; yara.shamikh@nub.edu.eg; 2 Consultant, Virology Department, Egypt Center for Research and Regenerative Medicine (ECRRM), Cairo 11517, Egypt; 3 Department of Microbiology and Public Health, Faculty of Pharmacy, Heliopolis University for Sustainable Development, Cairo 11785, Egypt; aliaa.ali@hu.edu.eg; 4 Department of Microbiology and Immunology, Faculty of Pharmacy, Beni-Suef University, Beni-Suef 62511, Egypt; yasser.gaber@pharm.bsu.edu.eg; 5 Department of Pharmaceutics and Pharmaceutical Technology, College of Pharmacy, Mutah University, Karak 61710, Jordan; 6 Department of Pharmacognosy, Faculty of Pharmacy, Minia University, Minia 61519, Egypt; usama.ramadan@mu.edu.eg; 7 Department of Pharmacognosy, Faculty of Pharmacy, Deraya University, 7 Universities Zone, New Minia 61111, Egypt; 8 Department of Pharmacognosy, College of Pharmacy, King Khalid University, Abha 61441, Saudi Arabia; 9 Department of Marine and Environmental Geology, National Institute of Oceanography and Fisheries, Red Sea Branch, Hurghada 84511, Egypt; madkour_hashem@yahoo.com; 10 Independent Researcher, 69126 Heidelberg, Germany; hannesdhorn@gmail.com; 11 Department of Pharmacognosy, Faculty of Pharmacy, Beni-Suef University, Beni-Suef 62511, Egypt; abuh20050@yahoo.com (H.M.H.); abeerabdelhakium@yahoo.com (A.H.E.); 12 Biology Department, College of Science, Princess Nourah Bint Abdulrahman University, Riyadh, Saudi Arabia; DHALkalifah@pnu.edu.sa; 13 Bioproducts Research Chair, Zoology Department, College of Science, King Saud University, Riyadh 11451, Saudi Arabia; 14 Botany and Microbiology Department, Faculty of Science, Beni-Suef University, Beni-Suef, Egypt

**Keywords:** sponges, actinomycetes, cryptic, *Micromonospora*, *Nocardia*, *Gordonia*, mycolic acid, LC-HRESIMS

## Abstract

The diversity of actinomycetes associated with the marine sponge *Coscinoderma mathewsi* collected from Hurghada (Egypt) was studied. Twenty-three actinomycetes were separated and identified based on the 16S rDNA gene sequence analysis. Out of them, three isolates were classified as novel species of the genera *Micromonospora*, *Nocardia*, and *Gordonia*. Genome sequencing of actinomycete strains has revealed many silent biosynthetic gene clusters and has shown their exceptional capacity for the production of secondary metabolites, not observed under classical cultivation conditions. Therefore, the effect of mycolic-acid-containing bacteria or mycolic acid on the biosynthesis of cryptic natural products was investigated. Sponge-derived actinomycete *Micromonospora* sp. UA17 was co-cultured using liquid fermentation with two mycolic acid-containing actinomycetes (*Gordonia* sp. UA19 and *Nocardia* sp. UA 23), or supplemented with pure mycolic acid. LC-HRESIMS data were analyzed to compare natural production across all crude extracts. *Micromonospora* sp. UA17 was rich with isotetracenone, indolocarbazole, and anthracycline analogs. Some co-culture extracts showed metabolites such as a chlorocardicin, neocopiamycin A, and chicamycin B that were not found in the respective monocultures, suggesting a mycolic acid effect on the induction of cryptic natural product biosynthetic pathways. The antibacterial, antifungal, and antiparasitic activities for the different cultures extracts were also tested.

## 1. Introduction

Actinomycetes are Gram-positive bacteria living in a wide range of aquatic, terrestrial environments and produce a variety of diverse bioactive compounds [1,2,3,4]. This phylum also has been found in a range of marine organisms such as corals, sponges, and jellyfish [5,6,7]. Actinomycetes from the marine environment have been reported to produce most of the bioactive compounds identified from the marine ecosystems [8,9]. These compounds belong to a variety of classes including polyketides, alkaloids, fatty acids, peptides, and terpenes [10,11,12,13,14]. There are many potential bioactivities of these compounds ranging from antibacterial, antifungal and antiparasitic to antioxidant and immunomodulatory activities [15,16,17]. With advances in sequencing technologies, actinomycete genomes have revealed many biosynthetic genes that encode for natural products not observed under standard fermentation conditions [18,19,20,21,22]. Previous methods were used to induce cryptic metabolites including chemical, molecular, and biological elicitation [22,23,24,25,26,27,28,29]. Altering the fermentation conditions (pH, media composition, and temperature) using the “one strain many compounds” (OSMAC) approach has been used to induce silent or poor expressed metabolic pathways [30,31,32,33]. Co-cultivation of microbial strains is a widely known approach to induce significant changes in the microbial metabolomes [20].

Mycolic acids are high-molecular-weight *α*-branched, *β*-hydroxyl fatty acids, which are located in the cell wall of certain bacterial genera such as *Corynebacterium*, *Mycobacterium Nocardia*, *Rhodococcus*, and *Segniliparus* [34,35,36]. They play a major role in shaping the cell wall and protect against chemical substances [37,38]. The structure of each mycolic acid is thought to be genus-specific and differs in the length of the carbon chain [34,39]. For example, members of the genus *Corynebacterium* have C_50_-C_56_ and the genus *Rhodococcus* has been found to contain C_34_-C_52_. Onaka et al., 2011, reported the induction of a red pigment by *Streptomyces lividans TK23* after co-cultivation with living cells of the mycolic acid-containing bacterium *Tsukamurella pulmonis* TP-B0596 [40]. It was shown that the metabolite profiles of several *Streptomyces* strains were changed after co-cultivation with the mycolic acid-containing bacterium *T. pulmonis*. Combined culture of *S. endus* S-522 with *T. pulmonis* resulted in the identification of a novel antibiotic, alchivemycin A [40]. Recently, Hoshino *et al*., 2015, isolated the di- and tri-cyclic macrolactams niizalactams A–C from the co-culture of *Streptomyces* sp. NZ-6 and the mycolic acid-containing bacterium *Tsukamurella pulmonis* TP-B0596 [41]. Arcyriaflavin E, a new cytotoxic indolocarbazole alkaloid, was isolated by co-cultivation of mycolic acid-containing bacteria and *Streptomyces cinnamoneus* NBRC 13823 [42]. Chojalactones A–C, cytotoxic butanolides were isolated from the co-culture of *Streptomyces* sp. cultivated with mycolic acid-containing bacterium *Tsukamurella pulmonis* TP-B0596 [43]. These studies highlight the efficacy of the co-cultivation strategy with mycolic acid-containing bacteria, for the discovery of cryptic natural products. Interestingly, all of those studies co-cultivate mycolic acid-containing bacteria with terrestrial actinomycetes however the effect on marine actinomycetes to our knowledge is yet to be investigated.

In this study, isolates of novel species belonging to the genera *Micromonospora*, *Nocardia*, and *Gordonia* were identified, and the effect of pure mycolic acid and mycolic acid-containing bacteria actinomycete *Gordonia* sp. UA19 and *Nocardia* sp. UA 23 on the secondary metabolite production of sponge-derived actinomycete *Micromonospora* sp. UA17 was examined by analyzing using LC-HRMS/MS data via metabolomes tools. The antibacterial, antifungal, and antiparasitic activities for the different cultures extracts were also tested.

## 2. Materials and Methods

### 2.1. Description of the Area for Sponge Collection

**Figure 1:**
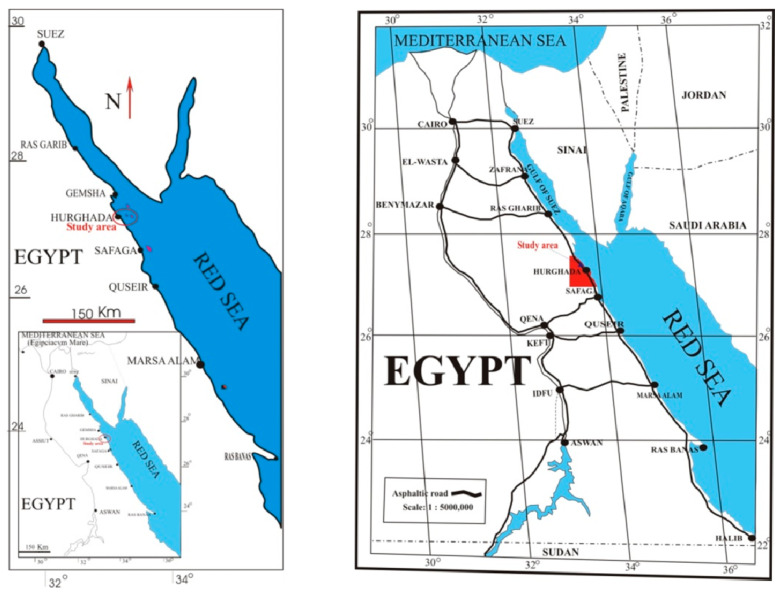
Location map of the study area along the Egyptian Red Sea coast.

The study area lies about 5 km to the north of Hurghada at latitudes 27°17’01.0” N, and longitudes 33°46’21.0” E (Figure 1). This site is characterized by a long patchy reef, representing the front edge of a wide and shallow reef flat with many depressions and lagoons. The depth ranged from about 3 m at the reef front with a gentle slope towards deep water. The area was exposed to strong waves, and the currents follow the prevailing current direction in the Red Sea from north to south. A medium development undergoes along the coast of this area. The bottom topography of this area is characterized by seagrasses and algae in intertidal and subtidal areas in addition to coral. The samples collected from this area, namely Ahia Reefs. *Coscinoderma mathewsi* was identified by El-Sayd Abed El-Aziz (Department of Invertebrates Lab., National Institute of Oceanography and Fisheries, Red Sea Branch, 84511 Hurghada, Egypt)

### 2.2. Chemicals and Reagents

All chemicals were of high analytical grade, purchased from Sigma Chemical Co Ltd. (St. Louis MO, USA).

### 2.3. Actinomycetes Isolation

The sponge biomass was transferred to the laboratory in a plastic bag containing seawater. Sponge specimens were washed with sterile seawater, cut into pieces of ~1 cm^3^, and then thoroughly homogenized in a sterile mortar with 10 volumes of sterile seawater. The supernatant was serially diluted (10^−1^, 10^−2^, 10^−3^) and subsequently plated onto agar plates. Three different media (M1, ISP2, and Marine Agar (MA)) were used for the isolation of actinomycetes. All media were supplemented with 0.2 µm pore size filtered cycloheximide (100 µg/mL), nystatin (25 µg/mL) and nalidixic acid (25 µg/mL) to facilitate the isolation of slow-growing actinomycetes. All media contained Difco Bacto agar (18 g/L) and were prepared in 1 L artificial seawater (NaCl 234.7 g, MgCl_2_.6 H_2_O 106.4 g, Na_2_SO_4_ 39.2 g, CaCl_2_ 11.0 g, NaHCO_3_ 1.92 g, KCl 6.64 g, KBr 0.96 g, H_3_BO_3_ 0.26 g, SrCl_2_ 0,24 g, NaF 0.03 g and ddH_2_O to 10.0 L). The inoculated plates were incubated at 30 °C for 6–8 weeks. Distinct colony morphotypes were picked and re-streaked until visually free of contaminants. *Micromonospora* sp. UA17, *Gordonia* sp. UA19 and *Nocardia* sp. UA 23 was cultivated on ISP2 medium. The isolates were maintained on plates at the fridge and in 20% glycerol at −80 °C.

### 2.4. Molecular Identification and Phylogenetic Analysis

The systematic position of the 16S rDNA sequences was analyzed with the SINA web aligner and the search and class option [44]. Closest relatives and type strains were obtained from GenBank using nucleotide Blast against nt and refseq_rna databases, respectively [45]. Alignments were calculated using again the SINA web aligner v1.2.11 (variability profile: bacteria). For maximum-likelihood tree construction RAxML v8.2.12 (-f a -m GTRGAMMA) was used with 100 bootstrap replicates [46]. Trees were visualized with interactive Tree of Life (iTol) v5.5 [47]. The 16S rDNA sequences of *Micromonospora* sp. UA17, *Nocardia* sp. UA23, and *Gordonia* sp. UA19 were deposited in GenBank under the accession numbers MT271359, MT271360, and MT271361.

### 2.5. Co-cultivation and Extract Preparation

Three actinomycetes were subjected to liquid fermentation as follows; each strain was fermented in 2 L Erlenmeyer flasks each containing 1.5 L ISP2 medium. After incubation of monocultures and co-cultures, the liquid cultures were grown for 10 days at 30 °C while shaking at 150 rpm. The culture was then filtered and the supernatant was extracted with ethyl acetate. The ethyl acetate extracts were stored at 4 °C. Mycolic acid was used at the concentration (5 µg/mL).

### 2.6. Metabolic Profiling

Ethyl acetate extracts from samples were prepared at 1 mg/mL for mass spectrometry analysis. The recovered ethyl acetate extract was subjected to metabolic analysis using LC-HR-ESI-MS according to Abdelmohsen et al. [33]. An Acquity Ultra Performance Liquid Chromatography system connected to a Synapt G2 HDMS quadrupole time-of-flight hybrid mass spectrometer (Waters, Milford, USA) was used. Positive and negative ESI ionization modes were utilized to carry out the high-resolution mass spectrometry coupled with a spray voltage at 4.5 kV, the capillary temperature at 320 °C, and mass range from *m/z* 150–1500. The MS dataset was processed and data were extracted using MZmine 2.20 based on the established parameters [48]. Mass ion peaks were detected and accompanied by chromatogram builder and chromatogram deconvolution. The local minimum search algorithm was addressed and isotopes were also distinguished via the isotopic peaks of grouper. Missing peaks were displayed using the gap-filling peak finder. An adduct search along with a complex search was carried out. The processed data set was next subjected to molecular formula prediction and peak identification. The positive and negative ionization mode data sets from the respective extract were dereplicated against the DNP (Dictionary of Natural Products) databases.

### 2.7. Mycolic Acid Detection

The existence of mycolic acid in the bacterial strains (*Gordonia* sp. UA19 and *Nocardia* sp. UA 23) was investigated following the protocol by Onaka et al., 2011 [40]. After 5–7 days fermentation, a broth culture (50 mL) was harvested and centrifuged (5000 rpm for 15 min), the resulting pellet was resuspended in 20 mL of 10% KOH-MeOH and then hydrolyzed by heating at 100 °C for 2 h. The solution was cooled to room temperature, and the hydrolyzed residues were acidified with 6N HCl and then extracted using *n*-hexane (30 mL). The hexane phase was collected and evaporated in vacuo. The residue was re-suspended in 20 mL of benzene-MeOH-H_2_SO_4_ (10:20:1) solution and incubated for 2 h at 100 °C. The solution was cooled to room temperature, and the esterified residue was extracted using 20 mL of water and *n*-hexane (1:1). Mycolic acid was obtained by concentrating on the *n*-hexane phase. To confirm the extraction procedure, a hexane phase aliquot was subjected to thin-layer chromatography (TLC) (silica gel 60 F254; Merck); using an *n*-hexane-diethyl ether (4:1) mobile phase, and then dipped in 50% H_2_SO_4_. The plates were heated at 150 °C, and the methyl ester derivatives of mycolic acid were detected as brown colored spots.

### 2.8. Antibacterial Activity

Antibacterial activity was tested against *Staphylococcus aureus* NCTC 8325, *Enterococcus faecalis*, *Escherichia coli* and *Pseudomonas aeruginosa* (Culture Collections Public Health England, Porton Down, UK) [49]. After 24 h incubation at 37 °C, broth cultures were diluted in Müller-Hinton broth (1:100) and cultivated again until the cells reached the exponential growth phase. Cells (10^5^ cells/mL) were incubated in the presence of various concentrations of the tested extracts in DMSO to the last volume of 200 µL in a 96-well plate at 37 °C. The final concentration of DMSO was 0.8% in each well. After 18 h of incubation, the optical density of the cultures was determined at 550 nm using an ELISA microplate reader (Dynatech Engineering Ltd., Willenhall, UK). The lowest concentration of the compound that inhibits bacterial growth was defined as the minimal inhibitory concentration (MIC), where chloramphenicol was used as a positive control (0.3 μg/mL).

### 2.9. Antifungal Activity

Antifungal activity was done by re-suspending a colony of *Candida albicans* 5314 (ATCC 90028) (Culture Collections Public Health England, Porton Down, UK) [50], in 2 mL of 0.9% NaCl. Four microliters of this suspension were transferred to 2 mL of HR medium. Various concentrations of the test extracts were diluted in 100 µL of a medium in a 96-well microplate with a final DMSO concentration of 0.4%. One hundred microliters of the *Candida* suspension were added to each well then incubated at 30 °C for 48 h. Optical density was measured at 530 nm for control well without *Candida* cells, and the MIC was detected. Amphotericin B was used as a positive control (MIC 0.4 μg/mL).

### 2.10. Anti-Trypanosomal Activity

The anti-trypanosomal activity was carried out according to the protocol of Huber and Koella using 104 trypanosomes per mL of *Trypanosoma brucei* brucei strain TC 221, which were cultivated in Complete Baltz Medium. Trypanosomes were tested in 96-well plate against different concentrations of test extracts at 10–200 µg/mL in 1% DMSO to a final volume of 200 µL. As a control, 1% DMSO and the parasite without the extract was used in each plate to show no effect of 1% DMSO. The plates were then incubated at 37 °C in an atmosphere of 5% CO_2_ for 24 h. After the addition of 20 µL of Alamar Blue, the activity was measured after 48 and 72 h by light absorption using an MR 700 Microplate Reader at a wavelength of 550 nm with a reference wavelength of 650 nm. The MIC values of the test extracts were quantified in by linear interpolation of three independent measurements. Suramin was used as a positive control (MIC 0.23 μg/mL).

### 2.11. Statistical Analysis

All experiments were carried out in triplicate. The data were presented as the means ± standard error of the mean (SEM) of at least three independent experiments. The differences among various treatment groups were determined by ANOVA followed by Dunnett’s test using PASW Statistics^®^ version18 (Quarry Bay, Hong Kong). The difference of *p* < 0.05 considered statistically significant compared with a vehicle-treated control group and showed by a ^*^ symbol. The MIC values were determined using a nonlinear regression curve fitting analysis using GraphPad Prism software version 6 (La Jolla, CA, USA).

## 3. Results and Discussion

### 3.1. Molecular Identification and Phylogenetic Analysis

The actinomycete diversity of the Red Sea sponge *Coscinoderma mathewsi* was investigated. Twenty-three isolates were selected based on their cultural characteristics appearance. The 16S rDNA genes were sequenced, and the resulted sequences were blasted against the GenBank database. The isolates were found to belong to six different genera, *Gordonia, Kocuria, Nocardia*, *Micrococcus, Micromonospora*, and *Microbacterium*. Three new species (*Micromonospora* sp. UA17, *Gordonia* sp. UA19, and *Nocardia* sp. UA 23) were identified based on sequence similarities < 98.2%. The sequence similarities of the three isolates against the type strains ranging from 95.39% to 96.97% (Table 1, Table 2 and Table 3).

**Figure 2:**
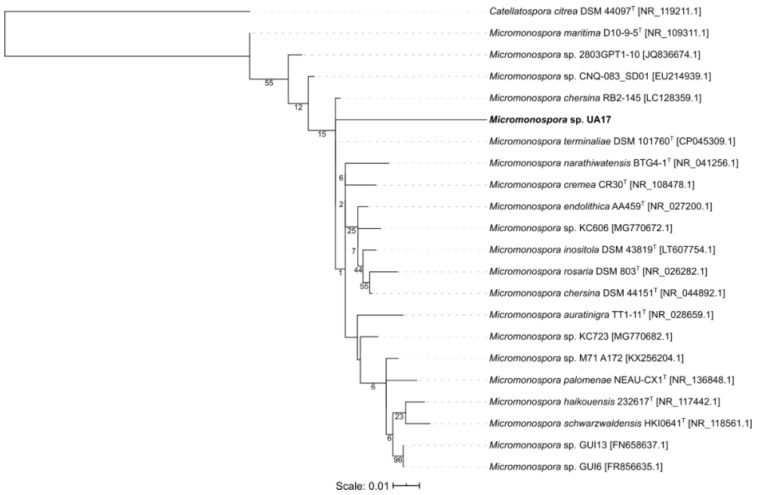
Maximum-likelihood tree of 21 *Micromonospora* representatives and one *Catellatospora* strain as an outgroup. Bootstrap values (100 resamples) are given in percent at the nodes of the tree. The isolate *Micromonospora* sp. UA17 obtained in this study is presented in bold.

**Figure 3:**
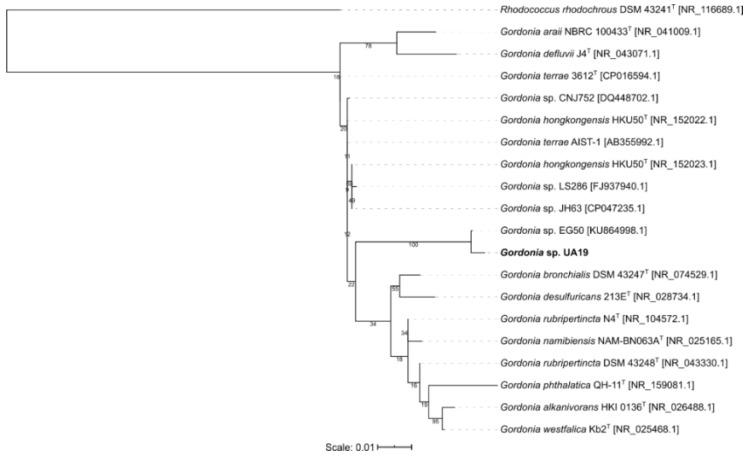
Maximum-likelihood tree of 19 *Gordonia* representatives and one *Rhodococcus* strain as an outgroup. Bootstrap values (100 resamples) are given in percent at the nodes of the tree. The isolate *Gordonia* sp. UA19 obtained in this study is presented in bold.

**Figure 4:**
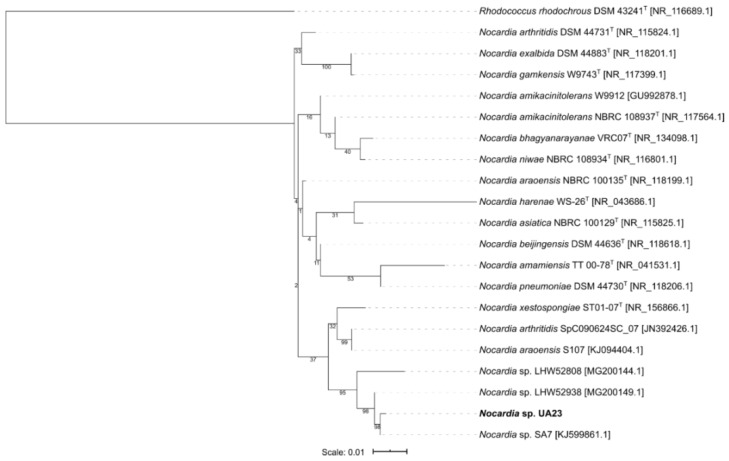
Maximum-likelihood tree of 20 *Nocardia* representatives and one *Rhodococcus* strain as an outgroup. Bootstrap values (100 resamples) are given in percent at the nodes of the tree. The isolate *Nocardia* sp. UA23 obtained in this study is presented in bold.

The phylogenetic tree for *Micormonospora* reveals the type strain *Micromonospora terminaliae* DSM 101760 to be the closest to *Micromonospora* sp. UA27 but did not show a specific cluster (Figure 2). The isolate *Gordonia* sp. UA19 was shown to be closest to the strain *Gordonia* sp. EG50, originally isolated from a marine sponge in the Red Sea and both seem to form an own cluster next to the obtained type strains (Figure 3). Isolate *Nocardia* sp. UA23 was placed with three strains, also isolated from marine sponges in either the Red Sea or the South China Sea (Figure 4).

It has been noticed that the discovery and isolation of new secondary metabolites are becoming hard tasks, as many of the gene clusters encoding for proteins involved in the production of these compounds are normally silenced under lab cultivation conditions [22,65]. To activate these biosynthetic pathways, several strategies have been developed [66,67]. One of the most effective ones consists of the co-cultivation of different microorganisms. The fermentation of both microorganisms in a common environment creates a competitive interaction between them. This fight for survival may induce the synthesis of secondary metabolites that have a defending function against the other microorganism present in the culture medium, thus resulting in the silent activation of gene clusters. Another efficient method for inducing the production of cryptic secondary metabolites is the use of elicitors [48,67,68]. Elicitors are molecules that unregulate the expression of gene clusters involved in the biosynthesis of secondary metabolites in bacteria and fungi.

Sponge-derived actinomycete *Micromonospora* sp. UA17 was co-cultured using liquid fermentation with two mycolic acid-containing actinomycetes (*Gordonia* sp. UA19 and *Nocardia* sp. UA 23) or supplemented with pure mycolic acid. The crude extracts were tested against bacteria, fungi, and the human parasite *Trypanosoma brucei.*

### 3.2. Metabolomic Profiling of Monoculture and Co-Culture Crude Extracts

Metabolite profiles from crude extracts of the actinobacterial monoculture extracts (*Micromonospora* sp. UA17, *Gordonia* sp. UA19 and *Nocardia* sp. UA 23), besides co-cultures with two strains of mycolic acid-containing bacteria (*Gordonia* sp. UA19 and *Nocardia* sp. UA 23) and monocultures amended with mycolic acid were analyzed. The existence of mycolic acid in the selected strains was confirmed as described by Onaka et al., 2011 [40]. The richest metabolites (in terms of several metabolites produced) were observed when the strain *Micromonospora* sp. UA17 was co-cultured with *Nocardia* sp. UA 23 (which contains mycolic acid) or when supplemented with mycolic acid.

#### 3.2.1. Chemical Dereplication of Micromonospora sp. UA17

Analyzing the *Micromonospora* sp. UA17, several hits were proposed (Supplementary Table S1, Supplementary Figure S1a). The molecular ion mass peaks at *m*/*z* 467.1350021, and 451.1401825 [M-H]^+^, for the predicted molecular formulas C_25_H_24_O_9_ and C_25_H_24_O_8_ gave hits of the isotetracenone type antitumor antibiotics atramycin A (**1**), and B (**2**) (Supplementary Figure S1b) that were previously isolated from *Streptomyces atratus* [69]. The mass ion peak at *m/z* 465.1557922, corresponding to the suggested molecular formula C_27_H_22_N_4_O_4_ [M-H]^+^ fit an antibiotic indolocarbazole derivative compound TAN-1030A (**3**) that was previously isolated from *Streptomyces longisporoflavus* R-19 [70]. The ion mass peak at *m/z* 323.091309 [M+H]^+^ for the predicted molecular formulas C_19_H_14_O_5_ gave hits of the anthracyclinone antibiotic fujianmycin A (**4**) which was isolated from *Streptomyces* sp. GW71/2497 [71]. Two major ion peaks with the *m/z* values of 529.171692 and 543.187439 [M-H]^+^ with molecular formulas C_27_H_30_O_11_ and C_28_H_32_O_11_ were detected and dereplicated as anthracycline antibiotic mutactimycin C (**5**) and A (**6**), respectively, which were isolated earlier from *Streptomyces* sp. 1254 [72]. In addition, the mass ion peaks at *m*/*z* 479.171387 [M-H]^+^, for the predicted molecular formula C_28_H_24_N_4_O_4_ was dereplicated another antibiotic indolocarbazole derivative 7-Oxostaurosporine (**7)**, which was previously detected in *Streptomyces platensis,* and reported as an inhibitor of protein kinase C (Figure S1b) [73].

#### 3.2.2. Chemical Dereplication of Gordonia sp. UA19

Analyzing the *Gordonia* sp. UA19, few hits were proposed (Supplementary Table S2, Supplementary Figure S2a). The molecular ion mass peak at *m*/*z* 199.0866394 [M+H]^+^ for the predicted molecular formulas C_12_H_10_N_2_O_2_ gave hits of the 5-(3-indolyl)oxazole type antiviral pimprinine (**8**) (Supplementary Figure S2b) that were previously isolated from *Streptomyces pimprina* [74] and reported as inhibitors against the replication of EV71 and ADV-7 [75]. Other than indolocarbazole derivative founded in *Micromonospora* sp. UA17, which had promising activities, heroin the mass ion peak at *m/z* 404.123993 which corresponded to the suggested molecular formula C_22_H_17_N_3_O_5_ [M+H]^+^ fit an indenotryptoline compound-cladoniamide C (**9**) that was previously isolated from *Streptomyces uncialis* [76], and yet showed no activity. Additionally, atramycin A (**1**), B (**2**), and fujianmycin A (**4**) metabolites were also dereplicated based on the mass ion peaks and in agreement with the molecular formulas.

#### 3.2.3. Chemical Dereplication of Nocardia sp. UA 23

When analyzing the *Nocardia* sp. UA 23, several hits were proposed (Supplementary Table S3, Supplementary Figure S3a,b). The molecular ion mass peak at *m*/*z* 161.08075 [M+H]^+^ for the predicted molecular formula C_7_H_12_O_4_ gave hits of the carbasugar gabosine-B (**10**) that were previously isolated from *Streptomyces albus* [77] and reported as having DNA-binding properties [78]. The molecular ion mass peaks at *m*/*z* 397.093338 [M-H]^+^ for the predicted molecular formula C_22_H_14_N_4_O_4_ gave hits of the quinoline-5,8-diones type antitumor antibiotic lavendamycin (**11**) that was previously isolated from *Streptomyces lavendulae* C-22030 S [79]. The mass ion peak at *m/z* 300.049118, corresponding to the suggested molecular formula C_10_H_12_ClN_5_O_4_ [M-H]^+^ fits the purine derivative 2-chloroadenosine (**12**) that was previously isolated from *Streptomyces rishiriensis* 265, Sp-265 (FERM-p 5921) [80] and reported as having a suppression effect in seizure [81]. The molecular ion mass peaks at *m*/*z* 249.107941 [M+H]^+^ for the predicted molecular formula C_9_H_16_N_2_O_6_ gave hits of the amino acid antibiotic malioxamycin (**13**) that was previously isolated from *Streptomyces lydicus* [82] and reported as having a role in the management of *Streptococcus pneumoniae* infection [83]. In addition, the mass ion peaks at *m*/*z* 407.167511[M-H]^+^ for the predicted molecular formula C_16_H_28_N_2_O_10_ was dereplicated as Enkastine I (**14**), which was a glycopeptide derivative, and was previously detected in *Streptomyces albus* ATCC 21838 [84]. According to the literature, Enkastine I was reported as a potent inhibitor of the endopeptidase 24.11, with an IC_50_ of 1.8 × 10^−9^ M [84]. Likewise, the molecular formula C_14_H_18_N_2_O_5_ was characterized as the antitumor antibiotic benzodiazepine derivative chicamycin A (**15**), from the mass ion peak at *m*/*z* 293.1146317 [M-H]^+^, which was previously obtained from *Streptomyces albus* [85,86]. The mass ion peaks at *m*/*z* 279.0975189 [M+H]^+^ and 215.1289215 [M-H]^+^ in agreement with the predicted molecular formulas C_13_H_14_N_2_O_5_, and C_11_H_20_O_4,_ and were dereplicated as benzodiazepine antibiotic RK-1441B (**16**), and the aliphatic alcohol antidepressant ketalin (**17**), respectively. These metabolites have been isolated earlier from *Streptomyces griseus* [87], and *Streptomyces lavendulae* Tue 1668 [88,89], respectively. In addition, the mass ion peak at *m*/*z* 1058.672668 [M+H]^+^ for the predicted molecular formula C_54_H_95_N_3_O_17_ was dereplicated as macrolide derivative antibiotic copiamycin which has in vitro activity against *Candida albicans*, *Torulopsis glabrata*, and *Trichomonas vaginalis*. Lo (**18**) and was previously detected in *Streptomyces hygroscopicus var. crystallogenes* [90,91].

#### 3.2.4. Chemical Dereplication of Strains UA17 + UA19

Analyzing co-culture *Micromonospora* sp. UA17 with mycolic acid-containing *Gordonia* sp. UA19 strain, interestingly dereplicated several hits (Supplementary Table S4, Supplementary Figure S4a, S4b). The molecular ion mass peak at *m*/*z* 252.124252 [M-H]^+^ for the predicted molecular formula C_13_H_19_NO_4_ gave hits of the piperidine derivative MY 336a (**19**) that was previously isolated from *Streptomyces gabonae* [92]. The molecular ion mass peaks at *m*/*z* 533.1067098 [M-H]^+^ for the predicted molecular formula C_23_H_23_ClN_4_O_9_ gave hits of the *β*-lactam derivative antibiotic chlorocardicin (**20**) that was previously isolated from *Streptomyces spp.* [93], and reported as an inhibitor to peptidoglycan biosynthesis [93]. The molecular ion mass peaks at *m*/*z* 335.056519 [M-H]^+^ for the predicted molecular formula C_19_H_12_O_6_ gave hits of the benzo[a]anthracene derivative antibiotic WS 5995-A (**21**) that was previously isolated from *Streptomyces auranticolor* 5995 (FERM-p 5365) [94]. The molecular ion mass peaks at *m*/*z* 1044.656982 [M+H]^+^ for the predicted molecular formula C_53_H_93_N_3_O_17_ gave hits of the macrolide derivative Neocopiamycin A (**22**) that was previously isolated from *Streptomyces hygroscopicus var. crystallogenes* [95] and reported to be more active against Gram-positive bacteria and fungi but less toxic than copiamycin (**18**) [90]. The previously identified metabolites atramycin A (**1**), B (**2**), pimprinine (**8**), and copiamycin (**18**), were also dereplicated based on the mass ion peaks and in agree with the molecular formulas.

#### 3.2.5. Chemical Dereplication of Strains UA17 + UA23

Analyzing co-culture *Micromonospora* sp. UA17 with mycolic acid-containing *Nocardia* sp. UA 23 strain, dereplicated several hits (Supplementary Table S5, Supplementary Figure S5a,b). A compound at *m/z* 215.1390686 [M+H]^+^, corresponding to the suggested molecular formula C_10_H_18_N_2_O_3_ was dereplicated as Alkaloid derivative LL-BH-872*α* (**23**), which was formerly reported from *Streptomyces hinnulinus* [96]. The mass ion peaks at *m/z* 247.1076736, 276.170578, 280.082809, 224.0916748, and 261.0881348 for the predicted molecular formulas C_13_H_14_N_2_O_3_, C_15_H_21_N_3_O_2_, C_13_H_15_NO_6_, C_11_H_13_NO_4_, and C_13_H_14_N_2_O_4_ were dereplicated as benzodiazepine DC 81 (**24**), eserine Alkaloid (**25**), amino derivative 13-hydroxy-streptazolin (**26**), pyridine-piperidine derivative A 58365B (**27**), and also benzodiazepine antitumor antibiotic chicamycin B (**28**), respectively, which were previously detected in *Streptomyces roseiscleroticus* do-81 (FERM-p 6502) [97], *Streptomyces griseofuscus* [98], *Streptomyces chromofuscus* [99], *Streptomyces sp.* A1 [100], and *Streptomyces albus* [86], respectively. Whereas that at *m/z* 229.1546555, corresponding to the suggested molecular formula C_11_H_20_N_2_O_3_ was dereplicated as imidazolidine derivative Libramycin-A (**29**), which was formerly reported from *Streptomyces sp.* [101]. Likewise, the molecular formulas C_16_H_27_N_3_O_6_, and C_11_H_11_NO_5_ was characterized as *β*-propiolactone amino acid belactosin C (**30**), and dehydrodioxolide B alkaloids (**31**), from the mass ion peaks at *m/z* 356.183197, and 236.0565987, which was previously obtained from *Streptomyces sp.* KY11780 [102], and *Streptomyces tendae* [103], respectively. Moreover, the characteristic metabolites atramycin A (**1**), chicamycin A (**15**), and chlorocardicin (**20**) were also dereplicated based on the mass ion peaks and in agreement with the molecular formulas.

#### 3.2.6. Chemical Dereplication of Strain UA17 with Mycolic Acid

Analyzing co-culture *Micromonospora* sp. UA17 with mycolic acid, dereplicated several hits (Supplementary Table S6, Supplementary Figure S6a,b). A compound at *m/z* 213.102264, corresponding to the suggested molecular formula C_13_H_12_N_2_O was dereplicated as 5-(3-indolyl)oxazole derivative antiviral pimprinethine (**32)**, which was formerly reported from *Streptoverticillium olivoreticuli* [75,104]. The mass ion peaks at *m/z* 259.1089478, 190.049843, 227.1178055, and 238.0722198 for the predicted molecular formulas C_14_H_16_N_2_O_3_, C_10_H_7_NO_3_, C_14_H_14_N_2_O, and C_11_H_13_NO_5_ were dereplicated as diketopiperazine derivative maculosin (**33**), antioxidant 3-Hydroxyquinoline-2-carboxylic acid (**34**), benzodiazepine derivative antitumor antibiotic prothracarcin (**35**), and amino acid N-acetyl-3,4-dihydroxy-L-phenylalanine (**36**), respectively which were previously detected in *Streptomyces rochei 87051-3* [105], *Streptomyces cyaneofuscatus* M-157 [106,107], *Streptomyces umbrosus* [108,109], and *Streptomyces akiyoshiensis* ATCC13480 L127 mutants [110], respectively. Whereas that at *m/z* 303.1353149, corresponding to the suggested molecular formula C_16_H_20_N_2_O_4_ was dereplicated as benzodiazepine derivative antitumor antibiotic tomaymycin (**37**), which was formerly reported from *Streptomyces achromogenes-tomaymyceticus* [111,112]. Likewise, the molecular formulas C_16_H_25_N_7_O_4_S, C_25_H_29_N_3_O_10_S were characterized as purine derivative antibiotic cystocin (**38**), and *β*-lactam derivative deoxycephamycin B (**39**) from the mass ion peaks at *m/z* 410.1608276, and 564.1655884 which were previously obtained from *Streptomyces* sp. GCA0001 [113] and *Streptomyces olivaceus* SANK 60384 (NRRL 3851) [114,115], respectively. Moreover, the characteristic metabolites, ketalin (**17**), chlorocardicin (**20**), DC 81 (**24**), eserine (**25**), 13-hydroxy-streptazolin (**26**), chicamycin B (**28**), libramycin-A (**29**), belactosin C (**30**), dehydrodioxolide B (**31**), were also dereplicated based on the mass ion peaks and in agreement with the molecular formulas.

### 3.3. Antibacterial, Antifungal, and Anti-Trypanosomal Activities

In this investigation, the crude extracts of the actinobacterial monoculture extracts (*Micromonospora* sp. UA17, *Gordonia* sp. UA19, and *Nocardia* sp. UA 23), beside co-cultures with two strains of mycolic acid-containing bacteria (*Gordonia* sp. UA19, and *Nocardia* sp. UA 23) and monocultures amended with mycolic acid were evaluated for their antibacterial, antifungal, and anti-trypanosomal activities against *Staphylococcus aureus* NCTC 8325, *Escherichia coli, Pseudomonas aeruginosa, Candida albicans* 5314, and *Trypanosoma brucei TC 221,* respectively. The results showed that *Micromonospora* sp. UA17 co-cultured with the two strains of mycolic acid-containing bacteria (*Gordonia* sp. UA19, and *Nocardia* sp. UA 23) and monocultures amended with mycolic acid were more active against *Staphylococcus aureus* NCTC 8325, *Enterococcus faecalis*, and *Candida albicans* 5314 compared with monoculture extracts, where UA17 + UA23 had recorded the highest inhibition activities with MIC value of 4.2, 3.9, and 3.8 µg/mL, respectively (Table 4). However, no activities were detected against *Escherichia coli* and *Pseudomonas aeruginosa.* These results suggest the mycolic acid affected the induction of bacterial natural product biosynthetic pathways. All tested extracts showed low activity against *Trypanosoma brucei* TC 221 (MIC > 100 µg/mL), except *Nocardia* sp. UA 23 which recorded the highest inhibition activities with MIC value of 7.2 µg/mL (Table 4).

## 4. Conclusions

The rapidly growing number of actinomycete genome sequences highlighted their potential for biosynthesizing a plethora of natural products that are much higher than expected during classical laboratory conditions. Biological elicitation (co-cultivation) of actinomycetes is an effective strategy to provoke the expression of unexpressed or poorly expressed secondary metabolites and further increasing their chemical diversity. This study highlighted the effect of co-culture with mycolic acid-producing microorganisms or mycolic acid itself in the induction of the biosynthesis of many metabolites; although they are known or previously isolated, it was first highlighted by this species because of the effect of co-culturing or the elicitor mycolic acid. On the other hand, some peaks showed no hits during dereplication which suggests they may be new metabolites and need further investigation in scale-up fermentation. The induction of these metabolites qualitatively and/or quantitatively may be the attributed to the difference in biological activities. As in *Micromonospora* sp. UA17 co-cultures with the two strains of mycolic acid-containing bacteria (*Gordonia* sp. UA19, and *Nocardia* sp. UA 23), monocultures amended with mycolic acid were more active against *Staphylococcus aureus* NCTC 8325, *Enterococcus faecalis*, and *Candida albicans* 5314 compared with monoculture extracts, where UA17 + UA23 had recorded the highest inhibition activities with MIC value of 4.2, 3.9, and 3.8 µg/mL, respectively. These results suggest that mycolic acid affected the induction of bacterial natural product biosynthetic pathways. On the other hand, all tested extracts showed low activity against *Trypanosoma brucei* TC 221, except *Nocardia* sp. UA 23 which recorded the highest inhibition activity with an MIC value of 7.2 µg/mL.

## Figures and Tables

**Table 1:** List of validly published strains of genus Micromonospora. Identity calculated against strain Micromonospora sp. UA17.

Isolate	Accession ID	Identity [%]	Source	Ref
*Micromonospora terminaliae* DSM 101760	CP045309.1	96.678	Surface sterilized stem of Thai medicinal plant *Terminalia mucronata*	[51]
*Micromonospora inositola* DSM 43819	LT607754.1	96.263	forest soil	[52]
*Micromonospora cremea* CR30	NR_108478.1	96.258	rhizosphere of *Pisum sativum*	[53]
*Micromonospora rosaria* DSM 803	NR_026282.1	96.125	unknown	[54]
*Micromonospora palomenae* NEAU-CX1	NR_136848.1	96.055	Nymphs of stinkbug (*Palomena viridissima* Poda)	[55]

**Table 2:** List of validly published strains of genus Gordonia. Identity calculated against strain Gordonia sp. UA19.

Isolate	Accession ID	Identity [%]	Source	Ref
*Gordonia hongkongensis* HKU50	NR_152023.1	95.386	human blood culture	[56]
*Gordonia terrae* 3612	CP016594.1	95.320	soil	[57]
*Gordonia bronchialis* DSM 43247	NR_074529.1	94.470	human sputum	[58]
*Gordonia desulfuricans* 213E	NR_028734.1	94.412	soil	[59]
*Gordonia rubripertincta* DSM 43248	NR_043330.1	94.345	soil	[60]

**Table 3:** List of validly published strains of genus Nocardia. Identity calculated against strain Nocardia sp. UA23.

Isolate	Accession ID	Identity [%]	Source	Ref
*Nocardia xestospongiae* ST01-07	NR_156866.1	96.972	*Xestospongia sp.*	[61]
*Nocardia amikacinitolerans* NBRC 108937	NR_117564.1	96.972	human eye (clinical isolate)	[62]
*Nocardia arthritidis* DSM 44731	NR_115824.1	96.898	human sputum	[63]
*Nocardia araoensis* NBRC 100135	NR_118199.1	96.677	human	[64]
*Nocardia beijingensis* DSM 44636	NR_118618.1	96.529	mud from a sewage ditch	NA

**Table 4:** Results of the crude extracts of the actinobacterial monoculture extracts (*Micromonospora* sp. UA17, *Gordonia* sp. UA19, and *Nocardia* sp. UA 23), beside co-cultures with two strains of mycolic acid-containing bacteria (*Gordonia* sp. UA19, and *Nocardia* sp. UA 23) and monocultures amended with mycolic acid against *Staphylococcus aureus* NCTC 8325, *Candida albicans* 5314, and *Trypanosoma brucei* TC 221.

Sample Code	MIC (µg/mL)			MIC (µg/mL, 72 h.) *Trypanosoma brucei* TC 221
	*Staphylococcus aureus* NCTC 8325	*Enterococcus faecalis*	*Candida albicans* 5314	
*Micromonospora* sp. UA17	15.6	14.3	13.2	>100
*Gordonia* sp. UA19	35.7	31.9	16.8	>100
*Nocardia* sp. UA 23	38.9	39.2	25.7	7.2 *
UA17 + UA19	8.6 *	7.4 *	6.4 *	>100
UA17 + UA23	4.2 *	3.9 *	3.8 *	>100
UA17 + Myc	4.7 *	3.8 *	5.9 *	>100

MIC value of compounds against tested the microorganism, which was defined as minimal inhibitory concentration (MIC). Data were expressed as mean ± 212 S.E.M (*n* = 3). One-way analysis of variance (ANOVA) followed by Dunnett’s test was applied. Graph Pad Prism 5 was used for statistical calculations (Graph pad Software, San Diego, CA, USA). * Significant (*p* < 0.05).

## Supplementary Materials

The following are available online at https://www.mdpi.com/2076-2607/8/5/783/s1.
