# Supplementary material for: Actinomycetes from the Red Sea Sponge Coscinoderma mathewsi: Isolation, Diversity, and Potential for Bioactive Compounds Discovery
Source: Microorganisms. 2020 May 23;8(5):783. doi: 10.3390/microorganisms8050783 (PMC7285244; doi:10.3390/microorganisms8050783)
Supplement: Supplementary file 1 [file microorganisms-08-00783-s001.pdf]

microorganisms

SUPPLEMENTARY MATERIAL

List of Contents

**Table S1.**	Dereplicated metabolites from LC-HRESIMS analysis of ***Micromonospora sp.*** **UA17**
**Table S2.**	Dereplicated metabolites from LC-HRESIMS analysis of ***Gordonia sp.*** **UA19**
**Table S3.**	Dereplicated metabolites from LC-HRESIMS analysis of ***Nocardia sp.*** **UA 23**
**Table S4.**	Dereplicated metabolites from LC-HRESIMS analysis of strains UA17 + UA19
**Table S5.**	Dereplicated metabolites from LC-HRESIMS analysis of strains **UA17 + UA23**
**Table S6.**	Dereplicated metabolites from LC-HRESIMS analysis of strains **UA17 + mycolic acid**
**Figure S1a**.	LC-HRESIMS Chromatogram of the dereplicated metabolites of *Micromonospora sp.* UA17
**Figure S2a**.	LC-HRESIMS Chromatogram of the dereplicated metabolites of *Gordonia sp.* UA19
**Figure S3a**.	LC-HRESIMS Chromatogram of the dereplicated metabolites of *Nocardia sp.* UA 23
**Figure S4a**.	LC-HRESIMS Chromatogram of the dereplicated metabolites of UA17 + UA19
**Figure S5a**.	LC-HRESIMS Chromatogram of the dereplicated metabolites of UA17 + UA23
**Figure S6a**.	LC-HRESIMS Chromatogram of the dereplicated metabolites of UA17 + mycolic acid
**Figure S1b**.	Dereplicated metabolites from LC-HRESIMS analysis of ***Micromonospora*** **sp. UA17**
**Figure S2b**.	Dereplicated metabolites from LC-HRESIMS analysis of ***Gordonia sp.*** **UA19**
**Figure S3b**.	Dereplicated metabolites from LC-HRESIMS analysis of ***Nocardia sp.*** **UA 23**
**Figure S4b**.	Dereplicated metabolites from LC-HRESIMS analysis of strains **UA17 + UA19**
**Figure S5b**.	Dereplicated metabolites from LC-HRESIMS analysis of strains **UA17 + UA23**
**Figure S6b**.	Dereplicated metabolites from LC-HRESIMS analysis of strains **UA17 + mycolic acid**

Table S1. Dereplicated metabolites from LC-HRESIMS analysis of ***Micromonospora sp.* UA17**

No.	Metabolite Name	Original Source	MF	Rt (min.)	MW	MS (*m/z*)
**1**	Atramycin A	*Streptomyces atratus*	$C_{25}H_{24}O_9$	8.28	468.141492	467.1350021
**2**	Atramycin B	*Streptomyces atratus*	$C_{25}H_{24}O_8$	9.68	452.146577	451.1401825
**3**	TAN-1030A	*Streptomyces longisporoflavus R-19*	$C_{27}H_{22}N_4O_4$	12.87	466.163565	465.1557922
**4**	Fujianmycin A	*Streptomyces* sp. GW71/2497	$C_{19}H_{14}O_5$	13.71	322.083584	323.091309
**5**	Mutactimycin C	*Streptomyces* sp. 1254	$C_{27}H_{30}O_{11}$	13.91	530.178272	529.171692
**6**	Mutactimycin A	*Streptomyces* sp. 1254	$C_{28}H_{32}O_{11}$	16.21	544.193922	543.187439
**7**	7-Oxostaurosporine	*Streptomyces platensis subsp.*	$C_{28}H_{24}N_4O_4$	20.62	480.179215	479.171387

MF: molecular formula, Rt: retention time, *m/z*: mass-to-charge ratio, min: minute

Table S2. Dereplicated metabolites from LC-HRESIMS analysis of ***Gordonia sp.* UA19**

No.	Metabolite Name	Original Source	MF	Rt	MW	MS (*m/z*)
**8**	Pimprinine	*Streptomyces pimprina*	$C_{12}H_{10}N_2O$	5.94	198.078773	199.0866394
**9**	Cladoniamide C	*Streptomyces uncialis*	$C_{22}H_{17}N_3O_5$	17.91	403.116281	404.123993

MF: molecular formula, Rt: retention time, *m/z*: mass-to-charge ratio, min: minute

Table S3. Dereplicated metabolites from LC-HRESIMS analysis of ***Nocardia sp.* UA 23**

No.	Metabolite Name	Original Source	MF	Rt	MW	MS (*m/z*)
**10**	Gabosine-B	*Streptomyces albus*	$C_7H_{12}O_4$	1.27	160.073019	161.08075
**11**	Lavendamycin	*Streptomyces lavendulae* C-22030 S	$C_{22}H_{14}N_4O_4$	1.40	398.100965	397.093338
**12**	2-chloroadenosine	*Streptomyces rishiriensis 265, Sp-265 (FERM-p 5921)*	$C_{10}H_{12}ClN_5O_4$	1.51	301.057242	300.049118
**13**	Malioxamycin	*Streptomyces lydicus*	$C_9H_{16}N_2O_6$	1.59	248.100296	249.107941
**14**	Enkastine I	*Streptomyces albus ATCC 21838*	$C_{16}H_{28}N_2O_{10}$	2.10	408.173855	407.167511
**15**	Chicamycin A	*Streptomyces albus*	$C_{14}H_{18}N_2O_5$	4.75	294.121031	293.1146317
**16**	RK-1441B	*Streptomyces griseus*	$C_{13}H_{14}N_2O_5$	6.01	278.089731	279.0975189
**17**	Ketalin	*Streptomyces lavendulae Tue 1668*	$C_{11}H_{20}O_4$	12.51	216.135619	215.1289215
**18**	Copiamycin	*Streptomyces hygroscopicus var. crystallogenes*	$C_{54}H_{95}N_3O_{17}$	31.11	1057.66560	1058.672668

MF: molecular formula, Rt: retention time, *m/z*: mass-to-charge ratio, min: minute

Table S4. Dereplicated metabolites from LC-HRESIMS analysis of strains **UA17 + UA19**

No.	Metabolite Name	Original Source	MF	Rt	MW	MS (*m/z*)
**19**	MY 336a	*Streptomyces gabonae*	$C_{13}H_{19}NO_4$	13.94	253.130868	252.124252
**20**	Chlorocardicin	*Streptomyces spp.*	$C_{23}H_{23}ClN_4O_9$	15.89	534.114816	533.1067098
**21**	WS 5995-A	*Streptomyces auranticolor* 5995 (FERM-p 5365)	$C_{19}H_{12}O_6$	19.49	336.062848	335.056519
**22**	Neocopiamycin A	*Streptomyces hygroscopicus var. crystallogenes*	$C_{53}H_{93}N_3O_{17}$	30.30	1043.64995	1044.656982

MF: molecular formula, Rt: retention time, *m/z*: mass-to-charge ratio, min: minute

Table S5. Dereplicated metabolites from LC-HRESIMS analysis of strains **UA17 + UA23**

No.	Metabolite Name	Original Source	MF	Rt	MW	MS (*m/z*)
**23**	LL-BH-872α	*Streptomyces hinnulinus*	$C_{10}H_{18}N_2O_3$	3.14	214.131202	215.1390686
**24**	DC 81	*Streptomyces roseiscleroticus do-81 (FERM-p 6502)*	$C_{13}H_{14}N_2O_3$	3.38	246.099902	247.1076736
**25**	Eserine	*Streptomyces griseofuscus*	$C_{15}H_{21}N_3O_2$	3.72	275.162837	276.170578
**26**	13-Hydroxy-streptazolin	*Streptomyces sp. A1*	$C_{11}H_{13}NO_4$	3.85	223.083918	224.0916748
**27**	A 58365B	*Streptomyces chromofuscus*	$C_{13}H_{15}NO_6$	4.11	281.089397	280.082809
**28**	Chicamycin B	*Streptomyces albus*	$C_{13}H_{14}N_2O_4$	4.18	262.094817	261.0881348
**29**	Libramycin-A	*Streptomyces sp.*	$C_{11}H_{20}N_2O_3$	5.03	228.146852	229.1546555
**30**	Belactosin C	*Streptomyces sp. KY11780*	$C_{16}H_{27}N_3O_6$	5.98	357.189445	356.183197
**31**	Dehydrodioxolide B	*Streptomyces tendae*	$C_{11}H_{11}NO_5$	6.14	237.063182	236.0565987

MF: molecular formula, Rt: retention time, *m/z*: mass-to-charge ratio, min: minute

Table S6. Dereplicated metabolites from LC-HRESIMS analysis of strains **UA17 + mycolic acid**

No.	Metabolite Name	Original Source	MF	Rt	MW	MS (*m/z*)
**32**	Pimprinethine	*Streptoverticillium olivoreticuli*	$C_{13}H_{12}N_2O$	3.22	212.094423	213.102264
**33**	Maculosin	*Streptomyces rochei 87051-3*	$C_{14}H_{16}N_2O_3$	3.75	260.115552	259.1089478
**34**	3-Hydroxyquinoline-2-carboxylic acid	Actinomycete *Streptomyces cyaneofuscatus* M-157	$C_{10}H_7NO_3$	4.01	189.042053	190.049843
**35**	Prothracarcin	*Streptomyces umbrosus*	$C_{14}H_{14}N_2O$	4.31	226.110073	227.1178055
**36**	N-Acetyl-3,4-dihydroxy-L-phenylalanine	*Streptomyces akiyoshiensis ATCC13480 L127 mutans*	$C_{11}H_{13}NO_5$	5.15	239.078832	238.0722198
**37**	Tomaymycin	*Streptomyces achromogenes-tomaymyceticus*	$C_{16}H_{20}N_2O_4$	5.61	304.141767	303.1353149
**38**	Cystocin	*Streptomyces sp. GCA0001*	$C_{16}H_{25}N_7O_4S$	9.09	411.168333	410.1608276
**39**	Deoxycephamycin B	*Streptomyces olivaceus SANK 60384 (NRRL 3851)*	$C_{25}H_{29}N_3O_{10}S$	15.32	563.156825	564.1655884

MF: molecular formula, Rt: retention time, *m/z*: mass-to-charge ratio, min: minute

microorganisms

MDPI

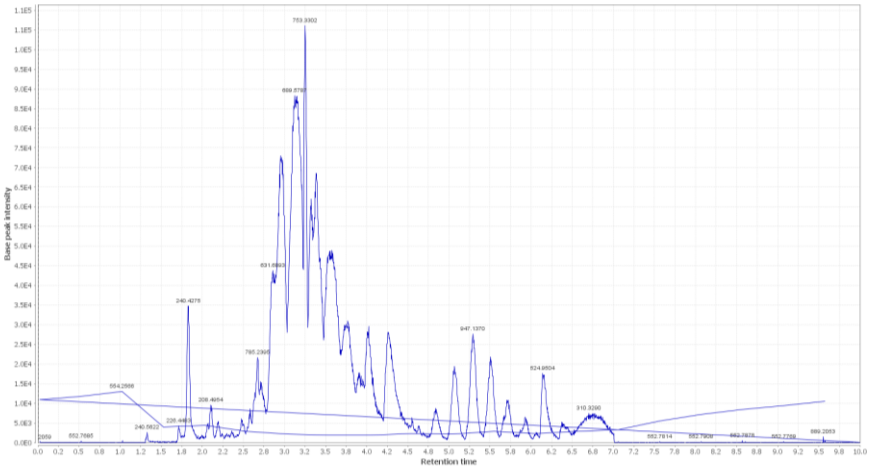

Figure S1a. LC-HRESIMS Chromatogram of the dereplicated metabolites of *Micromonospora sp.* UA17

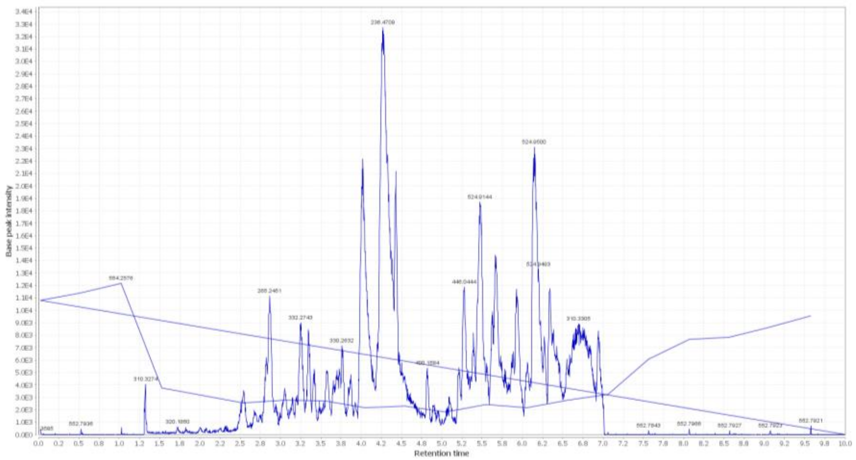

Figure S2a. LC-HRESIMS Chromatogram of the dereplicated metabolites of *Gordonia sp.* UA19

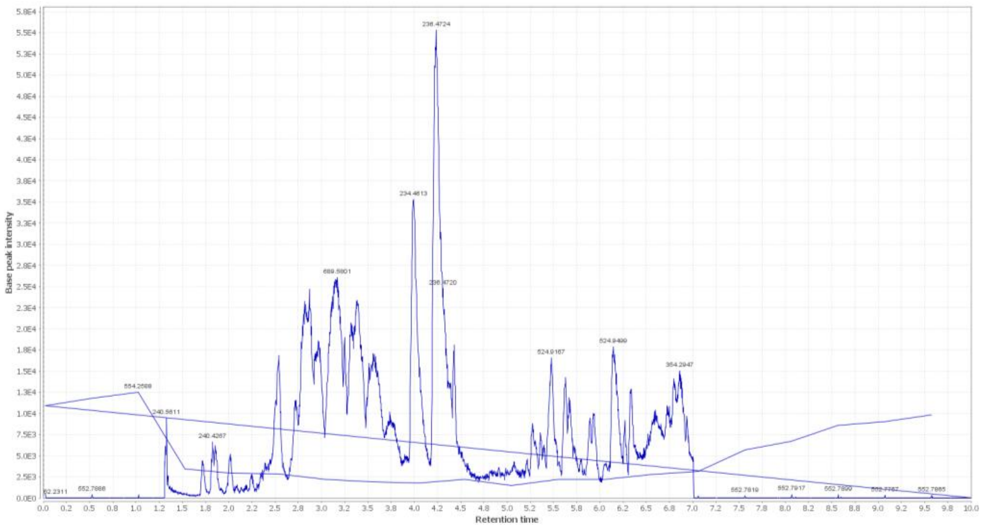

Figure S3a. LC-HRESIMS Chromatogram of the dereplicated metabolites of *Nocardia sp.* UA 23

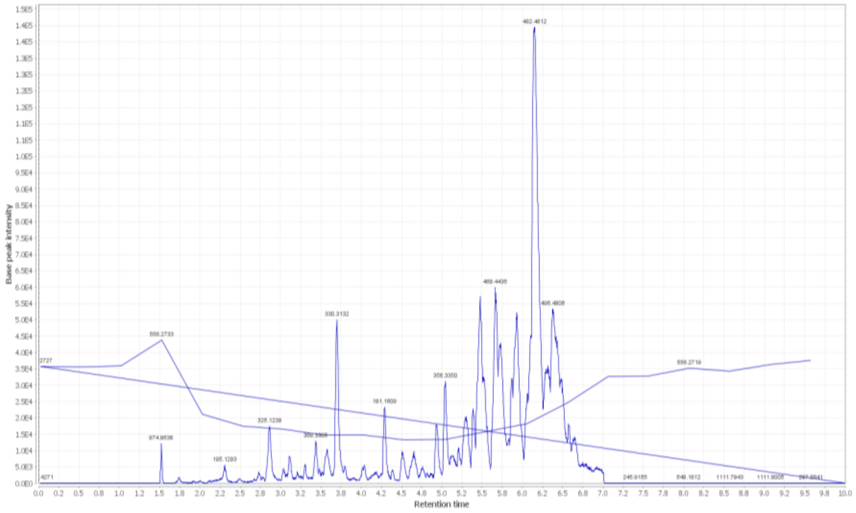

Figure S4a. LC-HRESIMS Chromatogram of the dereplicated metabolites of UA17 + UA19

microorganisms

MDPI

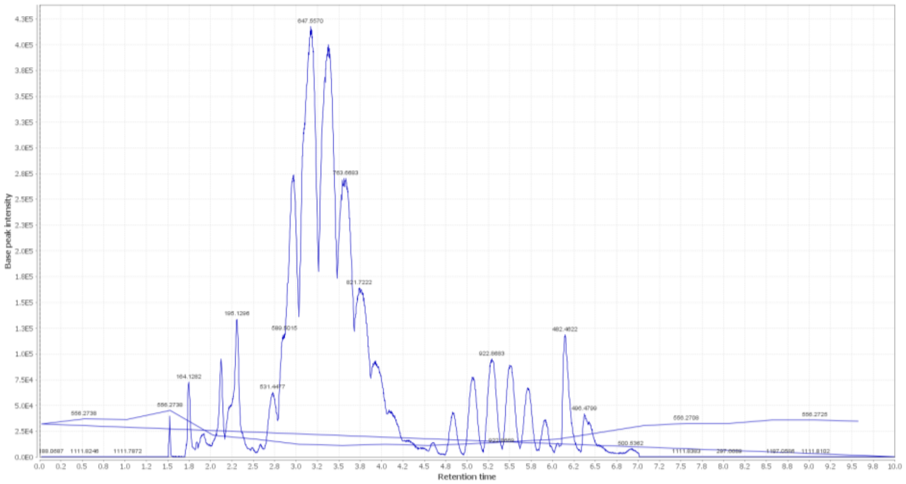

Figure S5a. LC-HRESIMS Chromatogram of the dereplicated metabolites of UA17 + UA23

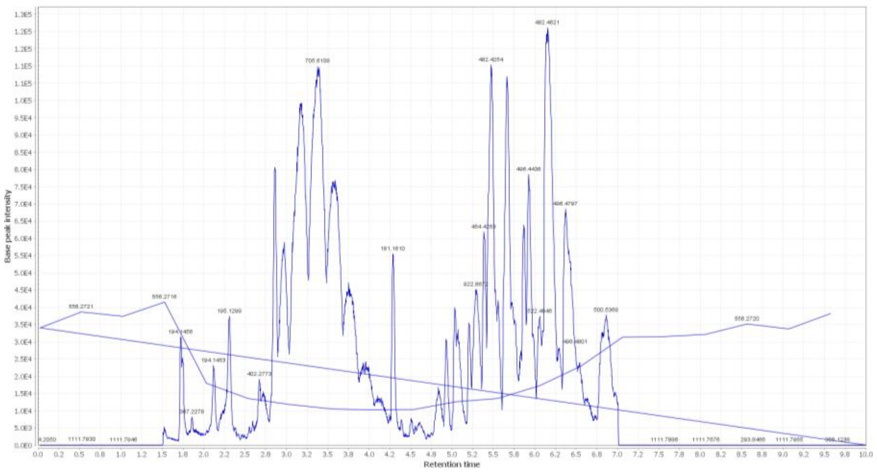

Figure S6a. LC-HRESIMS Chromatogram of the dereplicated metabolites of UA17 + mycolic acid

microorganisms
MDPI

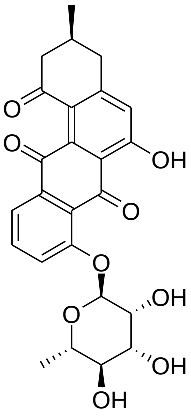

Atramycin A **(1)**

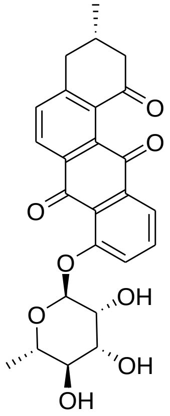

Atramycin B **(2)**

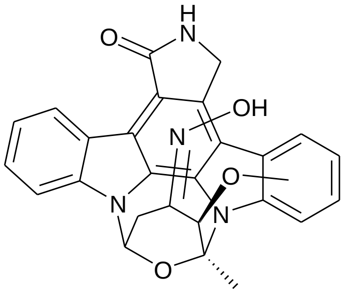

TAN-1030A **(3)**

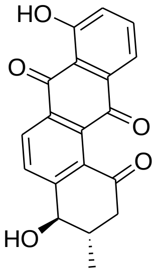

Fujianmycin A **(4)**

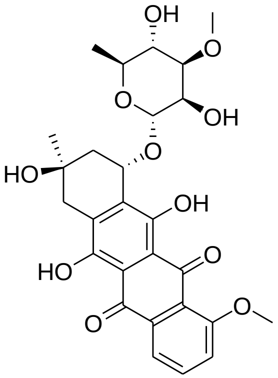

Mutactimycin C **(5)**

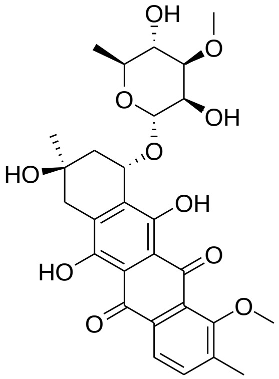

Mutactimycin A **(6)**

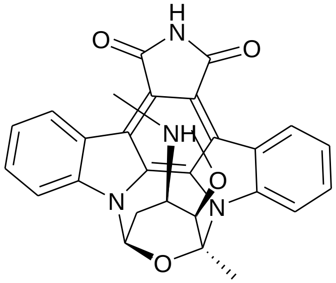

7-Oxostaurosporine **(7)**

Figure S1b. Dereplicated metabolites from LC-HRESIMS analysis of ***Micromonospora* sp. UA17**

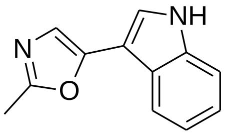

Pimprinine **(8)**

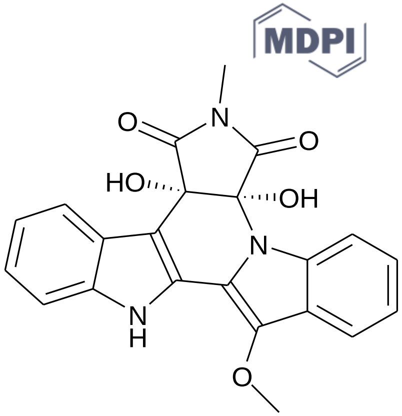

Cladoniamide C **(9)**

Figure S2b. Dereplicated metabolites from LC-HRESIMS analysis of ***Gordonia sp.* UA19**

microorganisms

MDPI

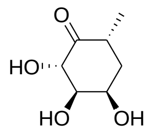

Gabosine-B **(10)**

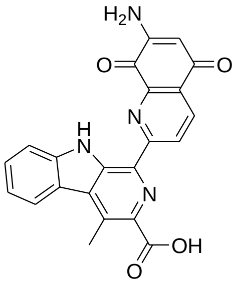

Lavendamycin **(11)**

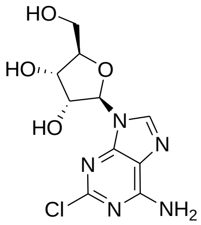

2-Chloroadenosine (**12**)

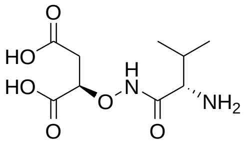

Malioxamycin **(13)**

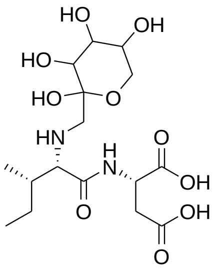

Enkastine I (**14**)

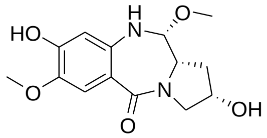

Chicamycin A **(15)**

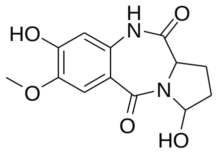

RK-1441B **(16)**

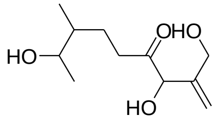

Ketalin (**17**)

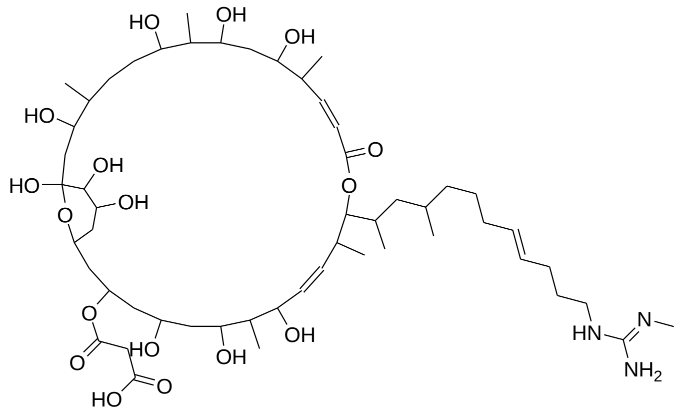

Copiamycin **(18)**

Figure S3b. Dereplicated metabolites from LC-HRESIMS analysis of ***Nocardia sp.*** **UA 23**

microorganisms

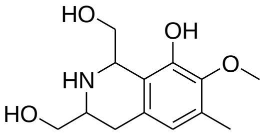

MY 336a **(19)**

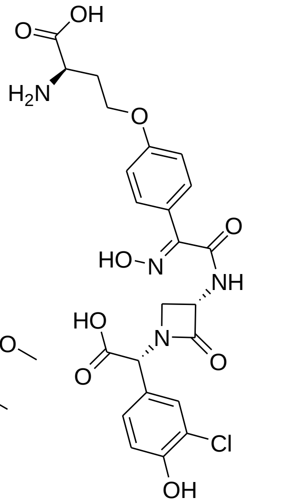

Chlorocardicin **(20)**

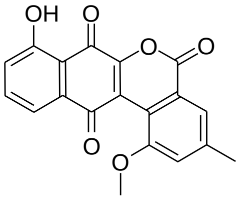

WS 5995-A **(21)**

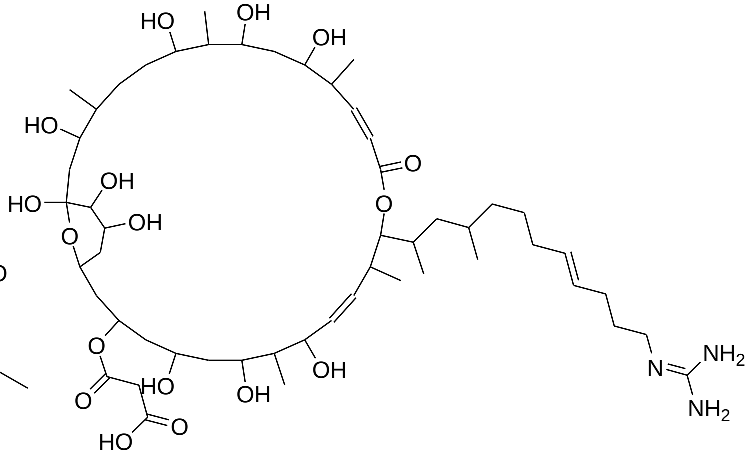

Neocopiamycin A **(22)**

Figure S4b. Dereplicated metabolites from LC-HRESIMS analysis of strains **UA17 + UA19**

microorganisms

MDPI

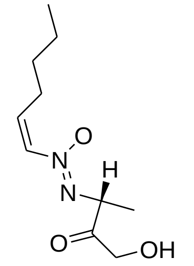

LL-BH-872α (23)

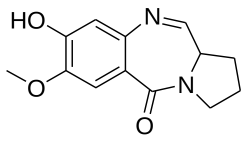

DC 81 **(24)**

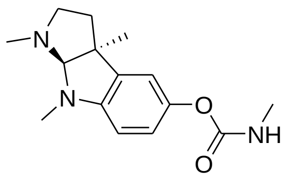

Eserine **(25)**

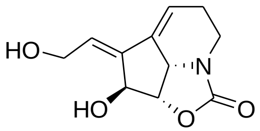

13-Hydroxy-streptazolin **(26)**

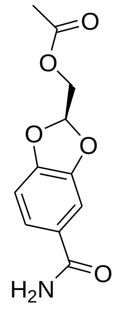

Dehydrodioxolide B **(31)**

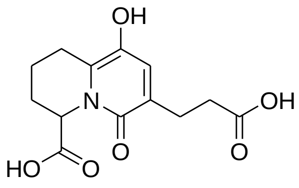

A 58365B **(27)**

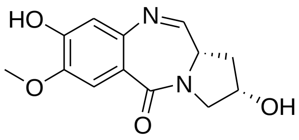

Chicamycin B **(28)**

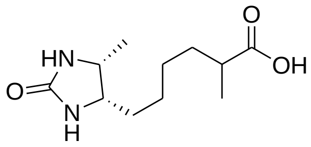

Libramycin-A **(29)**

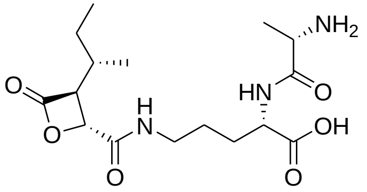

Belactosin C **(30)**

Figure S5b. Dereplicated metabolites from LC-HRESIMS analysis of strains **UA17 + UA23**

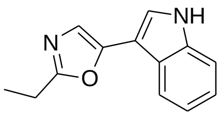

Pimprinethine **(32)**

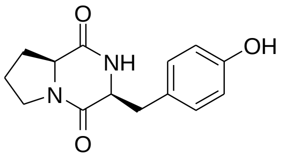

Maculosin **(33)**

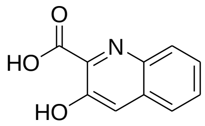

3-Hydroxyquinoline-2-carboxylic acid **(34)**

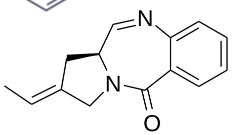

Prothracarcin **(35)**

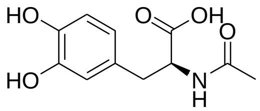

N-acetyl-3,4-dihydroxy-L-phenylalanine **(36)**

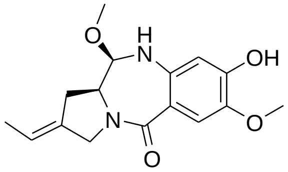

Tomaymycin **(37)**

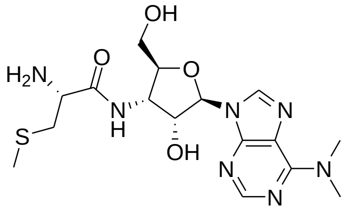

Cystocin **(38)**

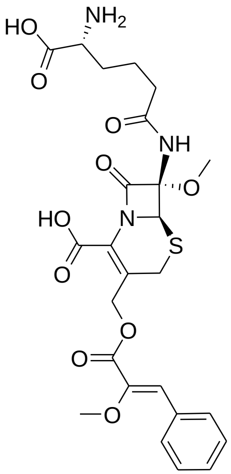

Deoxycephamycin B **(39)**

Figure S6b. Dereplicated metabolites from LC-HRESIMS analysis of strains **UA17 + mycolic acid**
